# Symptoms of COVID-19 contagion in different social contexts in association to self-reported symptoms, mental health and study capacity in Swedish university students

**DOI:** 10.1186/s13104-022-06009-z

**Published:** 2022-04-09

**Authors:** Claes Andersson, Marcus Bendtsen, Olof Molander, Lilian Granlund, Naira Topooco, Karin Engström, Petra Lindfors, Anne H. Berman

**Affiliations:** 1 Department of Criminology, Malmö University, Malmö, Sweden; 2 Department of Psychology, Uppsala University, Uppsala, Sweden; 3 Department of Health, Medicine and Caring Sciences, Linköping University, Linköping, Sweden; 4 Centre for Psychiatry Research, Department of Clinical Neuroscience, Karolinska Institutet, & Stockholm Health Care Services, Region Stockholm, Stockholm, Sweden; 5 Department of Behavioral Sciences and Learning, Linköping University, Linköping, Sweden; 6 Department of Global Public Health, Karolinska Institutet, Stockholm, Sweden; 7 Department of Psychology, Stockholm University, Stockholm, Sweden

**Keywords:** COVID-19, University students, Contagion in others, Self-contagion, Mental health, Study capacity

## Abstract

**Objective:**

The present study investigates if symptoms of COVID-19 contagion in different social contexts (cohabitants, family, acquaintances, and others) are associated with university students' own self-reported symptoms of COVID-19 contagion, mental health, and study capacity. This was investigated by a cross-sectional survey administrated in Sweden during the first wave of the COVID-19 pandemic, at the time when universities were locked down to limit viral spread and contagion.

**Results:**

Mild to moderate symptoms of COVID-19 in cohabitants and family members were associated with student’s self-reported symptoms of contagion, while no associations could be seen in relation to mental health and study capacity. Symptoms of COVID-19 contagion in acquaintances and others were not associated with students’ self-reported symptoms, nor with their mental health and study capacity.

To conclude, during the initial lockdown of universities students’ self-reported symptoms of contagion were mainly associated with cohabitants and family members, while symptoms of contagion in different social contexts were not associated with mental health and study capacity. Findings suggest that lockdown of universities may have contributed to limiting infection pathways, while still allowing students to focus on their studies despite significant contagion among others known to the student.

**Supplementary Information:**

The online version contains supplementary material available at 10.1186/s13104-022-06009-z.

## Introduction

After identification in late 2019, the COVID-19 virus spread across the world with ever increasing numbers of infected individuals [1] and excessive mortality rates [2]. In Sweden, the first wave lasted between March 19 to July 25, 2020 [3]. The wide range of somatic symptoms associated with the virus [4] resulted in uncertainty about whether individuals were infected [5]. Since the virus is transmitted through social contacts [6], measures were introduced to increase social distancing [7]. In March 2020, the Swedish government ordered universities into lockdown, with all education transferred to online learning to limit viral spread and contagion.

University students are recognized as a sensitive population with an increased risk of mental health problems, which has been shown to have a negative effect on study capacity [8]. During the pandemic-induced lockdown of universities, there have been several reports on negative mental health effects in university students [9–12]. To our knowledge, there are fewer reports on whether lockdowns have affected study results, though Berman et al. [11] report reduced academic self-efficacy. Similarly, limited research has been published so far on the question of the extent of COVID-19 spread among students. A Chinese review, not including university students, estimates that transmission primarily occurred within households [13], and a publication from the British Office for National Statistics [14] reports that transmission of the virus has been greater in halls of residence than in private housing.

It is widely recognized that social relationships have powerful effects on physical and mental health, and there are several theories on the relationship between social ties and health [15]. Recent findings suggest that knowing someone who has been infected with COVID-19 has a negative impact on student mental health [16], and that contagion among family members negatively affects both mental health and the ability to carry on with studies [17]. Since social relationships are important for both transmission of the virus and mental health, the current study intends to expand knowledge in the field.

## Objective of the present study

During the initial phase of the lockdown of Swedish universities, this study investigates whether symptoms of COVID-19 contagion in different social contexts (cohabitants, family, acquaintances, and others) are associated with university students' own symptoms of self-contagion, mental health, and study capacity.

## Main text

### Method

The present study was described in the original analysis plan [18]. Associations between compliance with public health recommendations and self-reported symptoms, mental health, and academic self-efficacy have previously been reported [11].

To investigate the effects of the COVID-19 pandemic in Swedish university students, a cross-sectional survey was distributed between May 18 and June 25, 2020. This was during the first wave of the pandemic, and at this time, university teaching had moved online. Voluntary response sampling was used. Participants were recruited through advertisements on the websites of 10 universities and that of the national association of student unions. Interested students followed an URL or QR-code to access detailed project information for informed consent, after which they accessed a short web survey. A total of 4495 students from 19 universities consented to participate. Of the respondents, 70.9% were women, with a mean age of 26.5 (SD 5.27).

The survey was developed for this specific study and the complete survey can be reviewed in a previous publication from the authors [19]. With reference to the importance of social relationship theories [15], the present study “focus” on associations between contagion in others and students’ reports on their own symptoms of self-contagion, mental health, and study capacity. The following four (1–4) questions were analyzed: (1) Respondents were asked to answer to what extent persons with whom they had been in contact had been tangibly affected by the COVID-19 pandemic. Responses for the following five (a-e) categories of persons were requested: (a) A person I live with; (b) A person in my family (not living with); (c) A person in my circle of acquaintances; (d) Someone else I have had contact with; (e) Another person. For each person, there were five (a-e) categorical response alternatives as follows: (a) No symptoms; (b) Mild symptoms; (c) Moderate symptoms; (d) Severe symptoms; Died; (e) Not relevant/Don’t know. (2) Respondents were asked to report whether they themselves had been affected in a tangible way by the COVID-19 pandemic by responding according to the following five (a-e) response alternatives: (a) No symptoms; (b) Mild symptoms; (c) Moderate symptoms; (d) Severe symptoms; (e) Not relevant/Don’t know. (3) The question of how mental health had been affected by the COVID-19 pandemic during the past 4 weeks had the following four (a-d) response alternatives: (a) No effect; (b) My mental health has been worse; (c) My mental health has been better; (d) My mental health has been both better and worse in different ways. (4) Finally, were students asked to rate “how their studies had been going” during the past 4 weeks by the following five (a-e) alternatives: (a) No change, my studies are going as usual; (b) My studies have been going worse; (c) My studies have been going better; (d) My studies have been going both better and worse in different ways; (e) I am not studying at this time.

The study objective was addressed by estimating associations among variables using multinomial regression. The salience of coefficients’ values with respect to the data was estimated using Bayesian inference with both standard normal priors and regularizing priors [20]. As several covariates were included in the model, the use of regularizing priors ensured a skeptical approach, pulling coefficients towards the null unless the data strongly suggested otherwise [21]. We complemented Bayesian analyses with maximum likelihood estimates and null-hypothesis testing. All models included age and gender as covariates.

### Results

Symptoms of contagion in self and in different social contexts are shown in Table 1 and self-reported effects on mental health and study capacity are shown in Table 2. It should be noted that about one third of the students reported having experienced symptoms of infection, and that self-reported symptoms were reported by the student respondents as prevalent among approximately 20% of their cohabitants and 25% of their non-cohabiting family members. Students generally reported that COVID-19 had affected their mental health and study capacity, with negative experiences being most prevalent.

**Table 1:** Symptoms of contagion in oneself and in different social contexts

	Total	None	Mild	Moderate	Severe	Died	Not relevant/Do not know
In oneself	4042	2025 (50.1)	931 (23.0)	419 (10.4)	66 (1.6)	–	601 (14.9)
Social context							
Cohabiting	3541	2090 (59.0)	470 (13.3)	179 (5.1)	20 (0.6)	0 (0.0)	782 (22.1)
Family	3560	2133 (59.9)	450 (12.6)	340 (9.6)	83 (2.3)	22 (0.6)	532 (14.9)
Acquaintance	3609	1266 (35.1)	725 (20.1)	892 (24.7)	236 (6.5)	60 (1.7)	430 (11.9)
Other, contact with	3528	1580 (44.8)	375 (10.6)	326 (9.2)	166 (4.7)	73 (2.1)	1008 (28.6)
Other	2589	1033 (39.9)	57 (2.2)	109 (4.2)	86 (3.3)	148 (5.7)	1156 (44.7)

Frequency (percent)

**Table 2:** Effects on mental health and study capacity

	Total	No effect/change	Worse	Better	Better & worse	Not studying
Mental health	3801	691 (19.2)	1661 (43.7)	293 (7.7)	1156 (30.4)	–
Study capacity	3726	471 (12.6)	1624 (43.6)	254 (6.8)	1289 (34.6)	88 (2.4)

Frequency (percent)

Multinomial regression analyses included complete cases for each outcome. The reference category for self-reported symptoms was No symptoms; for mental health it was No effects; and for study capacity it was also No effects. Table 3 and Additional file 1: Fig. S1 show that COVID-19 mainly spread in the circle of individuals close to respondents. Self-reports of mild symptoms were associated with mild and moderate symptoms in cohabitants, and with mild symptoms in family members. Self-reports of mild symptoms were also associated with uncertainty regarding contagion in cohabitants, family members, and acquaintances. Self-reports of moderate symptoms were associated with mild symptoms, moderate symptoms, and uncertainty regarding contagion in cohabitants. Self-reported uncertainty about infection was associated with uncertainty regarding contagion in cohabitants, family members, and acquaintances.

**Table 3:** Associations between contagion in different social contexts and self-reported symptoms

	Self-reported symptoms, n = 2529 (56.3%)								
	None	Mild		Moderate		Severe		Not relevant/do not know	
	n (%)	n (%)	% OR > 1, Median (95% CI)	n (%)	% OR > 1, Median (95% CI)	n (%)	% OR > 1, Median (95% CI)	n (%)	% OR > 1, Median (95% CI)
Cohabiting									
None	1445 (76.9)	311 (39.7)		105 (34.4)		21 (48.8)		208 (39.1)	
Mild	100 (5.3)	258 (33.0)	> 99.9, 9.22 (6.79; 10.27) *	79 (25.9)	> 99.9, 8.75 (5.71; 10.15) *	5 (11.6)	53.3, 1.00 (0.81; 1.02)	28 (5.3)	54.7, 1.00 (0.80, 1.05)
Moderate	33 (1.8)	65 (8.3)	> 99.9, 5.81 (3.49; 6.94) *	64 (21.0)	> 99.9, 18.07 (10.51; 21.79) *	3 (7.0)	54.3, 1.00 (0.82; 1.03)	14 (2.6)	59.5, 1.01 (0.81; 1.09)
Severe	3 (0.2)	8 (1.0)	76.5, 1.20 (0.78; 1.94)	4 (1.3)	63.1, 1.02 (0.71; 1.25)	4 (9.3)	60.9, 1.00 (0.85; 1.08)	1 (0.2)	43.1, 0.99 (0.37; 1.03)
Died	0 (0.0)	0 (0.0)	–	0 (0.0)	–	0 (0.0)	–	0 (0.0)	–
Not relevant/do not know	297 (15.8)	141 (18.0)	99.0, 1.55 (1.05; 1.75) *	53 (17.4)	98.6, 1.92 (1.02; 2.3) *	10 (23.3)	55.5, 1.00 (0.86–1.03)	281 (52.8)	> 99.9, 2.01 (1.41; 2.27) *
Family									
None	1343 (71.0)	387 (49.5)		164 (53.8)		21 (50.0)		218 (40.4)	
Mild	194 (10.3)	174 (22.3)	> 99.9, 2.19 (1.59; 2.44) *	35 (11.5)	26.7, 0.95 (0.54; 1.00)	9 (21.4)	60.1, 1.00 (0.88; 1.05)	38 (7.1)	34.6, 0.98 (0.65; 1.01)
Moderate	173 (9.1)	78 (10.0)	66.4, 1.03 (0.84; 1.14)	47 (15.4)	56.5, 1.01 (0.81; 1.07)	4 (9.5)	52.6, 1.00 (0.82; 1.02)	38 (7.1)	44.0, 1.00 (0.72; 1.02)
Severe	40 (2.1)	16 (2.0)	53.1, 1.00 (0.64; 1.11)	14 (4.6)	66.4, 1.03 (0.79; 1.22)	0 (0.0)	46.9, 1.00 (0.47; 1.01)	13 (2.4)	58.3, 1.01 (0.79; 1.09)
Died	9 (0.5)	3 (0.4)	37.7, 0.97 (0.34; 1.04)	3 (1.0)	53.1, 1.00 (0.63; 1.08)	1 (2.4)	49.1, 1.00 (0.56; 1.02)	6 (1.1)	64.7, 1.02 (0.80; 1.20)
Not relevant/do not know	133 (7.0)	124 (15.9)	> 99.9, 2.39 (1.55; 2.76) *	42 (13.8)	71.2, 1.04 (0.86; 1.20)	7 (16.7)	57.8, 1.00 (0.87; 1.04)	226 (41.5)	> 99.9, 3.28 (2.15; 3.78) *
Acquaintance									
None	793 (41.1)	232 (29.3)		104 (33.8)		17 (40.5)		120 (21.8)	
Mild	366 (19.1)	207 (26.1)	92.0, 1.20 (0.96; 1.34)	51 (16.6)	30.1, 0.97 (0.64; 1.00)	10 (23.8)	48.8, 1.00 (0.77; 1.02)	91 (16.5)	59.6, 1.01 (0.86; 1.06)
Moderate	482 (25.1)	195 (24.6)	50.2, 1.00 (0.82; 1.05)	94 (30.5)	58.0, 1.01 (0.84; 1.06)	4 (9.5)	32.4, 0.99 (0.23; 1.00)	117 (21.3)	36.8, 0.99 (0.74; 1.01)
Severe	126 (6.6)	50 (6.3)	46.8, 1.00 (0.71; 1.05)	20 (6.5)	32.4, 0.97 (0.50; 1.01)	3 (7.1)	46.0, 1.00 (0.59; 1.01)	37 (6.7)	60.6, 1.01 (0.84; 1.08)
Died	30 (1.6)	8 (1.0)	30.5, 0.93 (0.34; 1.01)	7 (2.3)	60.4, 1.02 (0.75; 1.15)	2 (4.8)	56.1, 1.00 (0.83; 1.03)	13 (2.4)	43.5, 0.99 (0.55; 1.03)
Not relevant/do not know	120 (6.3)	100 (12.6)	89.8, 1.28 (0.94; 1.51)	32 (10.4)	56.8, 1.01 (0.78; 1.07)	6 (14.3)	55.7, 1.00 (0.84; 1.03)	172 (31.3)	99.8, 2.02 (1.26; 2.32) *
Other, contact with									
None	989 (52.6)	317 (41.0)		135 (44.9)		18 (42.9)		121 (22.7)	
Mild	178 (9.5)	115 (14.9)	91.0, 1.24 (0.95; 1.43)	25 (8.3)	43.8, 0.99 (0.69; 1.03)	5 (11.9)	45.5, 1.00 (0.61; 1.01)	52 (9.8)	68.6, 1.02 (0.89; 1.13)
Moderate	177 (9.4)	65 (8.4)	64.9, 1.03 (0.83; 1.14)	32 (10.6)	43.0, 0.99 (0.67; 1.03)	3 (7.1)	52.1, 1.00 (0.79; 1.02)	49 (9.2)	65.1, 1.02 (0.88; 1.11)
Severe	86 (4.6)	38 (4.9)	47.9, 1.00 (0.68; 1.06)	14 (4.7)	26.8, 0.94 (0.36; 1.00)	1 (2.4)	48.5, 1.00 (0.69; 1.02)	27 (5.1)	81.8, 1.17 (0.92; 1.57)
Died	39 (2.1)	14 (1.8)	42.7, 0.99 (0.53; 1.05)	5 (1.7)	35.8, 0.98 (0.29; 1.02)	1 (2.4)	51.9, 1.00 (0.77; 1.02)	14 (2.6)	59.0, 1.01 (0.79; 1.09)
Not relevant/do not know	411 (21.9)	224 (29.0)	61.7, 1.02 (0.84; 1.10)	90 (29.9)	72.2, 1.05 (0.89; 1.19)	14 (33.3)	54.0, 1.00 (0.86; 1.02)	269 (50.6)	61.8, 1.01 (0.88; 1.07)
Other									
None	546 (41.6)	291 (47.5)		112 (47.5)		17 (50.0)		67 (17.0)	
Mild	28 (2.1)	14 (2.3)	23.8, 0.89 (0.40; 1.00)	7 (3.0)	61.6, 1.02 (0.77; 1.14)	0 (0.0)	46.5, 1.00 (0.41; 1.01)	8 (2.0)	47.4, 1.00 (0.65; 1.03)
Moderate	66 (5.0)	17 (2.8)	3.1, 0.55 (0.28; 0.71) *	10 (4.2)	35.5, 0.98 (0.47; 1.02)	2 (5.9)	51.6, 1.00 (0.77; 1.02)	14 (3.6)	54.2, 1.00 (0.78; 1.05)
Severe	52 (4.0)	15 (2.5)	17.8, 0.84 (0.40; 0.98)	4 (1.7)	36.0, 0.98 (0.41; 1.02)	0 (0.0)	45.2, 1.00 (0.24; 1.01)	15 (3.8)	55.9, 1.00 (0.79; 1.07)
Died	75 (5.7)	28 (4.6)	3.9, 0.60 (0.33; 0.76) *	9 (3.8)	18.6, 0.83 (0.26; 0.99)	1 (2.9)	46.0, 1.00 (0.62; 1.01)	35 (8.9)	91.2, 1.52 (0.97; 1.98)
Not relevant/do not know	546 (41.6)	247 (40.4)	< 0.01, 0.48 (0.34; 0.53) *	94 (39.8)	3.3, 0.65 (0.41; 0.78) *	14 (41.2)	45.9, 1.00 (0.71; 1.01)	255 (64.7)	80.4, 1.08 (0.94; 1.23)

Asterisk (*) indicates associations identified when applying regularizing priors

*n* frequency, *OR* Odds Ratio, *CI* Confidence Interval

Associations between contagion in different social contexts and self-reported mental health effects and self-reported study capacity are shown in Additional file 2: Tables S1 and Additional file 3: Table S2. The results show that there was no marked association between symptoms of contagion in different social contexts and students’ mental health and study capacity.

### Discussion

This study shows associations between students’ self-reported symptoms of COVID-19 contagion during lockdown of Swedish universities, and symptoms of contagion in cohabitants and family members. This finding is consistent with an early review by Lei et al. [13]. Nonetheless, a significant overall number of students reported experiencing symptoms of COVID-19. This lack of association between self-contagion and symptoms of contagion outside the close circle of cohabitants and family members, can be interpreted to be due to limited infection pathways through successful exercising of social distancing.

Considering previous research findings, reported by Alemany-Arrebola et al. [17] and Browning et al. [16], we were surprised to find that symptoms in the close circle of cohabitants and family members did not affect students' mental health and study capacity in Sweden. These differences can perhaps be explained methodologically and/or by country variations in student housing, lockdown of other part of the society and by available means for testing at the time. For instance, Alemany-Arrebola et al. [17] investigated levels of anxiety in relation to illness and death of a relative/friend due to COVID-19 in Spain while Browning et al. [16] combined negative emotions and anxiety into a psychological impact scale, which was associated with US students’ responding that they knew someone infected. It is possible that the high level of access to digitized services in Swedish society, combined with universities moving their teaching online, has allowed students to keep up their studies despite significant contagion among others known to the student.

We found that many students experienced uncertainties regarding their own and others' symptoms. In the spring of 2020, there were few publicly funded opportunities to test for COVID-19. The Public Health Agency of Sweden had, however, issued clear information on the symptoms associated with infection, including fever, coughing, and breathing difficulties; there was, however, some public awareness of asymptomatic cases, now known to be common. It should be noted that uncertainty regarding infection in others did not affect mental health or study capacity.

### Conclusion

There are two key findings of the present study: First, respondent-reported mild to moderate symptoms of COVID-19 in cohabitants and family members were associated with self-reports of contagion; Second, COVID-19 symptoms in different social contexts, both proximal and distal to the respondent, ranged from mild levels to outcomes of death and were not associated with negative effects on students’ mental health and study capacity. The practical implications of these findings are that lockdown of Swedish universities may have contributed to limiting infection pathways, and that students might have been able to continue focus on their studies despite significant contagion among individuals close to them. Based on these findings, future lockdown of universities could be useful to limit the spread of COVID-19 and other future mass viral infections.

### Strengths

The present study included students from about 40% of Swedish universities and response rates are equivalent to about 2.6% of registered students at these universities. Considering the current survey method designed for rapid implementation, these results should be considered satisfactory.

## Limitations

This study lacks objective measures regarding one's own and others' symptoms of COVID-19 contagion. At the time of the study, objective tests were largely lacking and uncertainty regarding the spread of infection was generally high in Sweden. It should be noted that the study examines self-reported symptoms and not confirmed contagion. A second limitation is use of single items to assess the intended constructs; in comparison to multiple item measures, single-item measures are considered to have low content validity, sensitivity, and reliability. However, single items can serve as a substitute for multiple-item measures since overall global single items correlate with multiple-item total scores. Additionally, single items are superior with respect to response burden and response error.

In comparison to official figures, women students are somewhat overrepresented. Also, it should be noted that women are generally more willing to follow COVID-19 public health recommendations [22]. Other limitations are self-report and a cross-sectional design. Our analytic approach is conservative, although self-reports carry a risk of common method variance with possible overestimation of associations [23].

## Supplementary Information

**Additional file 1:** **Figure S1.** Associations between contagion in different social contexts and self-reported symptoms.

**Additional file 2:** **Table S2.** Associations between contagion in different social contexts and self-reported mental health effects.

**Additional file 3: Table S3.** Associations between contagion in different social contexts and self-reported study capacity.

## Data Availability

The data generated or analyzed during the current study are available from the corresponding author on reasonable request.

## Abbreviations

SD: Standard Deviation
n: Population size
OR: Odds Ratio
CI: Confidence Interval
