# Supplementary material for: Symptoms of COVID-19 contagion in different social contexts in association to self-reported symptoms, mental health and study capacity in Swedish university students
Source: BMC Res Notes. 2022 Apr 9;15:131. doi: 10.1186/s13104-022-06009-z (PMC8994063; doi:10.1186/s13104-022-06009-z)
Supplement: Supplementary file 1 — Additional file 1: Figure S1. Associations between contagion in different social contexts and self-reported symptoms. [file 13104_2022_6009_MOESM1_ESM.docx]

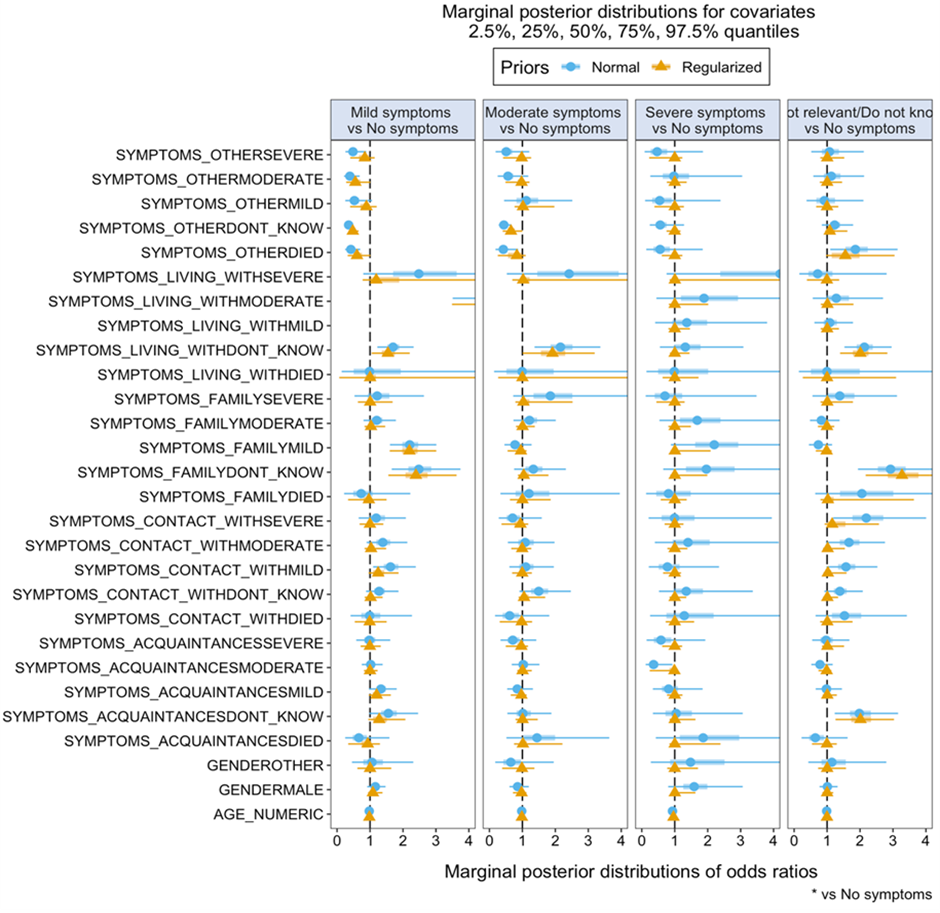


Supplementary Figure S1. Associations between contagion in different social contexts and self-reported symptoms.
