# Supplementary material for: Symptoms of COVID-19 contagion in different social contexts in association to self-reported symptoms, mental health and study capacity in Swedish university students
Source: BMC Res Notes. 2022 Apr 9;15:131. doi: 10.1186/s13104-022-06009-z (PMC8994063; doi:10.1186/s13104-022-06009-z)
Supplement: Supplementary file 2 — Additional file 2: Table S2. Associations between contagion in different social contexts and self-reported mental health effects. [file 13104_2022_6009_MOESM2_ESM.docx]

Supplementary Table S2. Associations between contagion in different social contexts and self-reported mental health effects.

|  |  | Self-reported mental health effects, n=2521 (56.1%) | | | | | | |
| --- | --- | --- | --- | --- | --- | --- | --- | --- |
|  |  | No effect | Worse | | Better | | Better & worse | |
|  |  | n (%) | n (%) | % OR > 1, Median (95% CI) | n (%) | % OR > 1, Median (95% CI) | n (%) | % OR > 1, Median (95% CI) |
| Cohabiting | None | 398 (63.9) | 898 (58.1) |  | 159 (58.7) |  | 623 (57.3) |  |
|  | Mild | 69 (11.1) | 226 (14.6) | 66.4, 1.01 (0.94; 1.05) | 41 (15.1) | 56.8, 1.00 (0.92; 1.02) | 134 (12.3) | 40.6, 0.99 (0.81; 1.01) |
|  | Moderate | 33 (5.3) | 78 (5.0) | 46.7, 1.00 (0.83; 1.01) | 13 (4.8) | 50.3, 1.00 (0.85; 1.01) | 55 (5.1) | 56.7, 1.00 (0.86; 1.04) |
|  | Severe | 0 (0.0) | 10 (0.6) | 55.5, 1.00 (0.84; 1.03) | 4 (1.5) | 52.2, 1.00 (0.84; 1.01) | 6 (0.6) | 47.4, 1.00 (0.62; 1.03) |
|  | Died | 0 (0.0) | 0 (0.0) | - | 0 (0.0) | - | 0 (0.0) | - |
|  | Not relevant/do not know | 123 (19.7) | 333 (21.6) | 53.4, 1.00 (0.90; 1.02) | 54 (19.9) | 51.8, 1.00 (0.89; 1.01) | 270 (24.8) | 79.7, 1.05 (0.95; 1.16) |
| Family | None | 405 (64.7) | 909 (58.6) |  | 163 (59.9) |  | 647 (59.0) |  |
|  | Mild | 64 (10.2) | 207 (13.3) | 64.4, 1.01 (0.93; 1.05) | 28 (10.3) | 46.0, 1.00 (0.82; 1.01) | 150 (13.7) | 45.3, 1.00 (0.84; 1.02) |
|  | Moderate | 49 (7.8) | 172 (11.1) | 75.3, 1.03 (0.95; 1.15) | 19 (7.0) | 41.3, 1.00 (0.64; 1.00) | 98 (8.9) | 44.0, 1.00 (0.80; 1.02) |
|  | Severe | 10 (1.6) | 39 (2.5) | 49.8, 1.00 (0.81; 1.02) | 11 (4.0) | 54.3, 1.00 (0.88; 1.02) | 23 (2.1) | 52.1, 1.00 (0.79; 1.04) |
|  | Died | 2 (0.3) | 9 (0.6) | 51.8, 1.00 (0.81; 1.02) | 2 (0.7) | 50.1, 1.00 (0.78; 1.01) | 8 (0.7) | 60.8, 1.01 (0.83; 1.09) |
|  | Not relevant/do not know | 96 (15.3) | 216 (13.9) | 53.9, 1.00 (0.90; 1.02) | 49 (18.0) | 67.7, 1.01 (0.95; 1.09) | 170 (15.5) | 42.9, 1.00 (0.81; 1.01) |
| Acquaintance | None | 273 (42.9) | 519 (32.9) |  | 86 (31.7) |  | 381 (34.4) |  |
|  | Mild | 128 (20.1) | 336 (21.3) | 67.5, 1.01 (0.94; 1.06) | 54 (19.9) | 51.4, 1.00 (0.89; 1.01) | 204 (18.4) | 16.5, 0.92 (0.67; 0.99) * |
|  | Moderate | 123 (19.3) | 408 (25.9) | 61.2, 1.00 (0.93; 1.04) | 70 (25.8) | 67.3, 1.01 (0.96; 1.08) | 287 (25.9) | 80.2, 1.05 (0.96; 1.16) |
|  | Severe | 31 (4.9) | 112 (7.1) | 70.9, 1.02 (0.94; 1.12) | 15 (5.5) | 45.3, 1.00 (0.76; 1.01) | 77 (6.9) | 59.6, 1.01 (0.87; 1.06) |
|  | Died | 10 (1.6) | 31 (2.0) | 62.9, 1.01 (0.90; 1.07) | 4 (1.5) | 50.8, 1.00 (0.82; 1.01) | 15 (1.4) | 31.2, 0.97 (0.36; 1.00) |
|  | Not relevant/do not know | 72 (11.3) | 172 (10.9) | 48.8, 1.00 (0.87; 1.02) | 42 (15.5) | 65.7, 1.00 (0.95; 1.07) | 144 (13.0) | 52.9, 1.00 (0.86; 1.03) |
| Other, contact with | None | 295 (47.4) | 688 (44.7) |  | 128 (47.8) |  | 461 (42.5) |  |
|  | Mild | 76 (12.2) | 168 (10.9) | 42.2, 1.00 (0.82; 1.01) | 27 (10.1) | 48.2, 1.00 (0.84; 1.01) | 103 (9.5) | 28.5, 0.97 (0.68; 1.00) |
|  | Moderate | 62 (10.0) | 130 (8.5) | 39.0, 1.00 (0.78; 1.01) | 28 (10.4) | 53.4, 1.00 (0.89; 1.01) | 105 (9.7) | 59.6, 1.01 (0.88; 1.05) |
|  | Severe | 24 (3.9) | 80 (5.2) | 61.7, 1.00 (0.91; 1.05) | 15 (5.6) | 53.3, 1.00 (0.88; 1.01) | 46 (4.2) | 46.7, 1.00 (0.78; 1.02) |
|  | Died | 15 (2.4) | 31 (2.0) | 59.1, 1.00 (0.89; 1.04) | 4 (1.5) | 47.2, 1.00 (0.71; 1.01) | 23 (2.1) | 50.6, 1.00 (0.76; 1.03) |
|  | Not relevant/do not know | 151 (24.2) | 441 (28.7) | 76.3, 1.03 (0.96; 1.13) | 66 (24.6) | 46.0, 1.00 (0.82; 1.01) | 347 (32.0) | 79.3, 1.06 (0.95; 1.18) |
| Other | None | 220 (45.7) | 466 (41.5) |  | 72 (37.1) |  | 271 (34.6) |  |
|  | Mild | 20 (4.2) | 22 (2.0) | 42.5, 1.00 (0.71; 1.01) | 4 (2.1) | 49.1, 1.00 (0.80; 1.01) | 11 (1.4) | 38.0, 0.99 (0.59; 1.01) |
|  | Moderate | 16 (3.3) | 43 (3.8) | 51.5, 1.00 (0.84; 1.02) | 5 (2.6) | 44.0, 1.00 (0.63; 1.01) | 45 (5.7) | 88.5, 1.32 (0.96; 1.68) |
|  | Severe | 24 (5.0) | 28 (2.5) | 28.9, 0.98 (0.50; 1.00) | 4 (2.1) | 44.8, 1.00 (0.69; 1.01) | 29 (3.7) | 58.1, 1.01 (0.85; 1.06) |
|  | Died | 19 (4.0) | 59 (5.3) | 41.6, 1.00 (0.78; 1.01) | 18 (9.3) | 63.1, 1.00 (0.94; 1.05) | 51 (6.5) | 76.2, 1.05 (0.93; 1.22) |
|  | Not relevant/do not know | 182 (37.8) | 505 (45.0) | 70.5, 1.01 (0.95; 1.08) | 91 (46.9) | 59.1, 1.00 (0.93; 1.02) | 376 (48.0) | 92.6, 1.20 (0.98; 1.34) |

Note. Asterisk (*) indicates associations identified when applying regularizing priors.

n = population size. OR = Odds Ratio. CI = Confidence Interval.
