# Supplementary material for: Symptoms of COVID-19 contagion in different social contexts in association to self-reported symptoms, mental health and study capacity in Swedish university students
Source: BMC Res Notes. 2022 Apr 9;15:131. doi: 10.1186/s13104-022-06009-z (PMC8994063; doi:10.1186/s13104-022-06009-z)
Supplement: Supplementary file 3 — Additional file 3: Table S3. Associations between contagion in different social contexts and self-reported study capacity. [file 13104_2022_6009_MOESM3_ESM.docx]

Supplementary Table S3. Associations between contagion in different social contexts and self-reported study capacity

|  |  | Self-reported change in study capacity, n=2482 (55.2%) | | | | | | | | |
| --- | --- | --- | --- | --- | --- | --- | --- | --- | --- | --- |
|  |  | No change | Worse | | Better | | Better & worse | | Not studying | |
|  |  | n (%) | n (%) | % OR > 1, Median (95% CI) | n (%) | % OR > 1, Median (95% CI) | n (%) | % OR > 1, Median (95% CI) | n (%) | % OR > 1, Median (95% CI) |
| Cohabiting | None | 260 (60.2) | 857 (56.9) |  | 145 (62.8) |  | 730 (60.0) |  | 48 (60.8) |  |
|  | Mild | 48 (11.1) | 213 (14.2) | 47.6, 1.00 (0.88; 1.02) | 32 (13.9) | 54.5, 1.00 (0.93; 1.01) | 161 (13.2) | 55.8, 1.00 (0.91; 1.02) | 12 (15.2) | 50.8, 1.00 (0.89; 1.01) |
|  | Moderate | 23 (5.3) | 87 (5.8) | 75.6, 1.04 (0.94; 1.24) | 15 (6.5) | 60.2, 1.00 (0.94; 1.02) | 45 (3.7) | 28.2, 0.98 (0.52; 1.00) | 5 (6.3) | 50.5, 1.00 (0.87; 1.01) |
|  | Severe | 0 (0.0) | 10 (0.7) | 57.9, 1.00 (0.84; 1.05) | 1 (0.4) | 50.7, 1.00 (0.84; 1.01) | 8 (0.7) | 49.1, 1.00 (0.75; 1.02) | 0 (0.0) | 48.7, 1.00 (0.78; 1.01) |
|  | Died | 0 (0.0) | 0 (0.0) | - | 0 (0.0) |  | 0 (0.0) | - | 0 (0.0) | - |
|  | Not relevant/do not know | 101 (23.4) | 338 (22.5) | 78.8, 1.04 (0.96; 1.13) | 338 (16.5) | 42.3, 1.00 (0.77; 1.00) | 273 (22.4) | 48.1, 1.00 (0.87; 1.01) | 14 (17.7) | 48.1, 1.00 (0.84; 1.01) |
| Family | None | 281 (65.2) | 888 (58.5) |  | 141 (60.3) |  | 735 (60.1) |  | 48 (61.5) |  |
|  | Mild | 43 (10.0) | 207 (13.6) | 73.3, 1.02 (0.95; 1.10) | 26 (11.1) | 46.0, 1.00 (0.83; 1.00) | 152 (12.4) | 45.4, 1.00 (0.86; 1.01) | 11 (14.1) | 52.8, 1.00 (0.91; 1.01) |
|  | Moderate | 33 (7.7) | 157 (10.3) | 66.2, 1.01 (0.93; 1.06) | 26 (11.1) | 54.8, 1.00 (0.92; 1.01) | 114 (9.3) | 36.5, 0.99 (0.75; 1.01) | 4 (5.1) | 43.9, 1.00 (0.66; 1.00) |
|  | Severe | 8 (1.9) | 41 (2.7) | 53.5, 1.00 (0.84; 1.03) | 5 (2.1) | 50.5, 1.00 (0.87; 1.01) | 25 (2.0) | 50.9, 1.00 (0.84; 1.02) | 1 (1.3) | 48.5, 1.00 (0.73; 1.01) |
|  | Died | 3 (0.7) | 8 (0.5) | 46.4, 1.00 (0.70; 1.02) | 2 (0.9) | 52.5, 1.00 (0.88; 1.01) | 6 (0.5) | 49.3, 1.00 (0.77; 1.02) | 0 (0.0) | 48.9, 1.00 (0.79; 1.01) |
|  | Not relevant/do not know | 63 (14.6) | 217 (14.3) | 58.8, 1.00 (0.91; 1.03) | 34 (14.5) | 54.0, 1.00 (0.92; 1.01) | 190 (15.5) | 63.6, 1.01 (0.93; 1.05) | 14 (17.9) | 52.7, 1.00 (0.92; 1.01) |
| Acquaintance | None | 181 (41.3) | 543 (35.3) |  | 78 (33.2) |  | 400 (32.3) |  | 32 (40.5) |  |
|  | Mild | 67 (15.3) | 313 (20.3) | 55.2, 1.00 (0.91; 1.02) | 53 (22.6) | 51.7, 1.00 (0.92; 1.01) | 262 (21.2) | 62.2, 1.00 (0.93; 1.04) | 15 (19.0) | 48.3, 1.00 (0.84; 1.01) |
|  | Moderate | 102 (23.3) | 387 (25.1) | 54.9, 1.00 (0.91; 1.02) | 45 (19.1) | 39.0, 1.00 (0.71; 1.00) | 318 (25.7) | 60.7, 1.00 (0.93; 1.03) | 20 (25.3) | 49.0, 1.00 (0.87; 1.01) |
|  | Severe | 27 (6.2) | 100 (6.5) | 66.6, 1.01 (0.93; 1.07) | 18 (7.7) | 51.7, 1.00 (0.90; 1.01) | 84 (6.8) | 42.7, 1.00 (0.79; 1.01) | 2 (2.5) | 46.0, 1.00 (0.59; 1.00) |
|  | Died | 9 (2.1) | 25 (1.6) | 50.4, 1.00 (0.81; 1.02) | 9 (3.8) | 57.3, 1.00 (0.93; 1.02) | 14 (1.1) | 44.1, 1.00 (0.93; 1.05) | 1 (1.3) | 49.6, 1.00 (0.84; 1.01) |
|  | Not relevant/do not know | 52 (11.9) | 171 (11.1) | 42.6, 1.00 (0.82; 1.01) | 32 (13.6) | 61.8, 1.00 (0.95; 1.03) | 160 (12.9) | 63.1, 1.00 (0.84; 1.01) | 9 (11.4) | 51.5, 1.00 (0.90; 1.01) |
| Other, contact with | None | 205 (47.6) | 688 (45.9) |  | 98 (42.2) |  | 516 (42.6) |  | 34 (43.6) |  |
|  | Mild | 32 (7.4) | 183 (12.2) | 63.9, 1.01 (0.93; 1.06) | 31 (13.4) | 53.2, 1.00 (0.92; 1.01) | 115 (9.5) | 45.4, 1.00 (0.84; 1.01) | 8 (10.3) | 46.1, 1.00 (0.76; 1.00) |
|  | Moderate | 45 (10.4) | 113 (7.5) | 3.6, 0.65 (0.43; 0.77) * | 29 (12.5) | 51.8, 1.00 (0.90; 1.01) | 123 (10.1) | 52.8, 1.00 (0.86; 1.02) | 9 (11.5) | 52.0, 1.00 (0.90; 1.01) |
|  | Severe | 26 (6.0) | 59 (3.9) | 37.6, 0.99 (0.70; 1.01) | 10 (4.3) | 49.4, 1.00 (0.87; 1.01) | 64 (5.3) | 53.5, 1.00 (0.88; 1.02) | 3 (3.8) | 48.4, 1.00 (0.83; 1.01) |
|  | Died | 11 (2.6) | 30 (2.0) | 50.2, 1.00 (0.81; 1.02) | 5 (2.2) | 45.8, 1.00 (0.65; 1.00) | 22 (1.8) | 49.6, 1.00 (0.82; 1.02) | 3 (3.8) | 50.6, 1.00 (0.88; 1.01) |
|  | Not relevant/do not know | 112 (26.0) | 427 (28.5) | 65.9, 1.01 (0.93; 1.06) | 59 (25.4) | 50.9, 1.00 (0.9; 1.01) | 372 (30.7) | 92.5, 1.21 (0.99; 1.37) | 21 (26.9) | 49.5, 1.00 (0.88; 1.01) |
| Other | None | 141 (42.3) | 466 (42.0) |  | 60 (35.9) |  | 317 (36.4) |  | 26 (43.3) |  |
|  | Mild | 7 (2.1) | 23 (2.1) | 50.8, 1.00 (0.82; 1.02) | 5 (3.0) | 51.1, 1.00 (0.89; 1.01) | 20 (2.3) | 53.5, 1.00 (0.87; 1.02) | 2 (3.3) | 49.7, 1.00 (0.86; 1.01) |
|  | Moderate | 13 (3.9) | 35 (3.2) | 31.1, 0.99 (0.58; 1.00) | 9 (5.4) | 50.8, 1.00 (0.89; 1.01) | 47 (5.4) | 73.9, 1.03 (0.95; 1.23) | 4 (6.7) | 50.9, 1.00 (0.87; 1.01) |
|  | Severe | 12 (3.6) | 29 (2.6) | 37.8, 0.99 (0.67; 1.01) | 4 (2.4) | 43.0, 1.00 (0.51; 1.00) | 37 (4.3) | 74.4, 1.04 (0.94; 1.29) | 2 (3.3) | 49.4, 1.00 (0.87; 1.01) |
|  | Died | 20 (6.0) | 53 (4.8) | 31.3, 0.98 (0.66; 1.00) | 16 (9.6) | 57.8, 1.00 (0.94; 1.02) | 53 (6.1) | 53.0, 1.00 (0.88; 1.02) | 4 (6.7) | 50.1, 1.00 (0.89; 1.01) |
|  | Not relevant/do not know | 140 (42.0) | 503 (45.4) | 64.6, 1.01 (0.94; 1.05) | 73 (43.7) | 50.2, 1.00 (0.91; 1.01) | 396 (45.5) | 56.7, 1.00 (0.92; 1.03) | 22 (36.7) | 45.0, 1.00 (0.80; 1.00) |

Note. Asterisk (*) indicates associations identified when applying regularizing priors.

n = population size. OR = Odds Ratio. CI = Confidence Interval.
